# Clopidogrel ameliorates high-fat diet-induced hepatic steatosis in mice through activation of the AMPK signaling pathway and beyond

**DOI:** 10.3389/fphar.2024.1496639

**Published:** 2024-10-23

**Authors:** Ting Tai, Yuan-Yuan Shao, Yu-Qi Zheng, Li-Ping Jiang, Hao-Ru Han, Na Yin, Hao-Dong Li, Jin-Zi Ji, Qiong-Yu Mi, Li Yang, Lei Feng, Fu-Yang Duan, Hong-Guang Xie

**Affiliations:** ^1^ Division of Clinical Pharmacology, General Clinical Research Center, Nanjing First Hospital, China Pharmaceutical University, Nanjing, China; ^2^ Department of Clinical Pharmacology, China Pharmaceutical University School of Basic Medicine and Clinical Pharmacy, Nanjing, China; ^3^ Division of Clinical Pharmacology, General Clinical Research Center, Nanjing First Hospital, Nanjing Medical University, Nanjing, China; ^4^ Department of Clinical Pharmacology, Nanjing Medical University School of Pharmacy, Nanjing, China

**Keywords:** AMPK, clopidogrel, fatty liver, hepatic steatosis, MASLD, NAFLD, steatotic liver

## Abstract

**Introduction:**

Metabolic dysfunction-associated steatotic liver disease (MASLD) frequently confers an increased risk of vascular thrombosis; however, the marketed antiplatelet drugs are investigated for the prevention and treatment of MASLD in patients with these coexisting diseases.

**Methods:**

To determine whether clopidogrel could ameliorate high-fat diet (HFD)-induced hepatic steatosis in mice and how it works, mice were fed on normal diet or HFD alone or in combination with or without clopidogrel for 14 weeks, and primary mouse hepatocytes were treated with palmitate/oleate alone or in combination with the compounds examined for 24 h. Body weight, liver weight, insulin resistance, triglyceride and total cholesterol content in serum and liver, histological morphology, transcriptomic analysis of mouse liver, and multiple key MASLD-associated genes and proteins were measured, respectively.

**Results and discussion:**

Clopidogrel mitigated HFD-induced hepatic steatosis (as measured with oil red O staining and triglyceride kit assay) and reduced elevations in serum aminotransferases, liver weight, and the ratio of liver to body weight. Clopidogrel downregulated the expression of multiple critical lipogenic (*Acaca*/*Acacb*, *Fasn*, *Scd1*, *Elovl6*, *Mogat1*, *Pparg*, *Cd36*, and *Fabp4*), profibrotic (*Col1a1*, *Col1a2*, *Col3a1*, C*ol4a1*, *Acta2*, and *Mmp2*), and proinflammatory (*Ccl2*, *Cxcl2*, *Cxcl10*, *Il1a*, *Tlr4*, and *Nlrp3*) genes, and enhanced phosphorylation of AMPK and ACC. However, compound C (an AMPK inhibitor) reversed enhanced phosphorylation of AMPK and ACC in clopidogrel-treated primary mouse hepatocytes and alleviated accumulation of intracellular lipids. We concluded that clopidogrel may prevent and/or reverse HFD-induced hepatic steatosis in mice, suggesting that clopidogrel could be repurposed to fight fatty liver in patients.

## Highlights

• Vascular thrombotic diseases and MASLD coexist in part of patients.
• Clopidogrel was used to determine whether it could ameliorate hepatic steatosis in mice.
• Clopidogrel downregulated multiple lipogenic, profibrotic, and proinflammatory genes.
• Clopidogrel significantly enhanced AMPK phosphorylation levels in the liver of mice.
• Clopidogrel may prevent and/or reverse hepatic steatosis in patients with MASLD.

## 1 Introduction

Metabolic dysfunction-associated steatotic liver disease (MASLD; formerly non-alcoholic fatty liver disease, or NAFLD) is currently the most prevalent chronic liver disease worldwide (Miao et al., 2024; Riazi et al., 2022), particularly in Asia (de Roza and Goh, 2019), which is comprised of a broad spectrum of progressive liver-associated disorders, including simple steatosis (or fatty liver), metabolic dysfunction-associated steatohepatitis (MASH; formerly non-alcoholic steatohepatitis, or NASH), hepatic fibrosis, cirrhosis, hepatocellular carcinoma (HCC), and even liver failure (Rinella et al., 2023; Sergi, 2024). As a multifactorial disease, MASLD is the result of the complicated interactions across obesity, insulin resistance, low-grade inflammation, and metabolic dysfunction (Sergi, 2024). Despite over 40-year efforts made globally, up to 14 March 2024, the US FDA approved the first drug – Razdiffra (resmetirom, its major active constituent) – for patients with liver scarring due to fatty liver disease (Anonymous, 2024). Considering that thyrotropin (TSH) increases triglyceride (TG) content in the liver through potentiation of sterol regulatory element-binding protein 1c (also known as SREBP-1c) activity (Yan et al., 2014) and *vice versa*, as an oral, liver-targeted, thyroid hormone receptor-β (THR-β) selective agonist, resmetirom was found to be superior to placebo for MASH resolution and improvement in liver fibrosis (Harrison et al., 2024). However, concomitant use of resmetirom and the statins (the lipid-lowering medications) might be warned (Anonymous, 2024).

In clinical settings, vascular thrombotic diseases, especially coronary artery disease, are common extrahepatic complications of MASLD (Miao et al., 2024; Semmler et al., 2023; Targher et al., 2010). In terms of the concept that an efficient approach to discovering a new drug is to start with an old drug (Xie et al., 2017), an old drug that has been marketed for coronary artery disease, such as the antiplatelet drugs, could be repurposed to combat MASLD if their pathogenesis is shared or linked. Earlier studies indicated that P2Y_12_ receptor expressed on the membrane of platelets is an important chemokine receptor, accelerating the secretion of the chemokines (such as Ccl2 and Cxcl2) through the phosphorylation of ERK1/2 and AKT upon ADP binding (Kloss et al., 2019), and that the activation of P2Y_12_ receptor decreases cholesterol efflux and inhibits autophagy of the vascular smooth muscle cells (VSMCs) via PI3K/AKT/mTOR signaling pathway (Pi et al., 2021). Conversely, blockade of the P2Y_12_ receptors ameliorates the accumulation of intracellular lipids and the formation of VSMC-derived foam cells through acceleration of autophagy of VSMCs in *ApoE* ^−/−^ mice when fed high-fat diet (HFD) (Pi et al., 2021) and suppresses the secretion of the chemokines (Kloss et al., 2019). Further, platelet-derived GPIbα was found to be a critical mediator and potential interventional target for the development of MASH and subsequent liver cancer, and antiplatelet therapy (aspirin/clopidogrel, and ticagrelor) may prevent the development of MASH and subsequent liver cancer through suppression of intrahepatic platelet accumulation and cytokine and chemokine release (Malehmir et al., 2019). Recently, antiplatelet therapy was proposed as a potential strategy for the treatment of MASLD (Boccatonda et al., 2024; Czajka et al., 2022; Fujita et al., 2008; Schwarzkopf et al., 2018), with some exception for hepatic steatosis (Malehmir et al., 2019), or clopidogrel (Lee et al., 2024). In terms of the fact that activation of adenosine monophosphate (AMP)-activated protein kinase (also known as AMPK) is a widely recognized common mechanism responsible for the treatment of MASLD (Trepiana et al., 2018; Zhu et al., 2018; 2019), that clopidogrel may activate CaMKKβ/AMPK/Nrf2 pathway (Yang et al., 2016), and that clopidogrel may effectively treat atherosclerosis through decreasing the number of cells positive for LRP1 (low-density lipoprotein receptor-related protein 1) and α-SMA (α-smooth muscle actin) in plaques from *ApoE* ^−/−^ mice fed an HFD (Chen et al., 2020), we hypothesized that clopidogrel could ameliorate HFD-induced hepatic steatosis in mice through enhancing phosphorylation of the AMPK pathway. To achieve this goal, we used HFD-induced obese mice to test this hypothesis and used primary mouse hepatocytes to elucidate the primary mechanisms involved.

## 2 Materials and methods

### 2.1 Study animals

All animal care and experimental procedures were approved by the Institutional Animal Care and Use Committee (IACUC), Nanjing Medical University (approval No. IACUC-2204054), China, and conducted in accordance with the Guide for the Care and Use of Laboratory Animals, issued by the US National Institutes of Health, as well as the ARRIVE guidelines (Lilley et al., 2020; Percie du Sert et al., 2020). In terms of the fact that the prevalence of MASLD is greater in adult men than in premenopausal women, and that estrogen is known to impart hepatic protection in women, especially at their premenopausal stages, most of the preclinical studies of MASLD were centered on using male mice as the primary subject of investigation (Burra et al., 2021; 2022; Gallage et al., 2022). In this study, only male mice were used because they may be more susceptible to MASLD, including but not limited to fatty liver (Gallage et al., 2022). Male C57BL/6J mice (aged 5–6 weeks old) were purchased from Jiangsu GemPharmatech Co., Ltd., Nanjing, China, and acclimated for 1 week before the study. All mice were housed within standardized SPF (specific pathogen-free) cages in a local, officially accredited experimental animal core facility at Nanjing Medical University, where the ambient temperature was maintained at 22°C–24°C, with a light to dark cycle alternated every 12 h. They had access to the required regular rodent chow diet (see below) and drinking water if needed but had been fasted for at least 6 h before being euthanized.

Male mice were randomly assigned to four groups: 1) normal diet (ND) group: mice fed a normal chow diet (cat #: 1010088; Jiangsu Xietong Rodent Diets Co. Ltd., Nanjing, China); 2) high-fat diet (HFD) group: mice fed an HFD of 60 kcal % fat (cat #: D12492; Research Diets, Inc., New Brunswick, United States); 3) HFD + low-dose clopidogrel group: mice fed HFD and drinking bottle water containing low-dose clopidogrel of 30 μg/mL, equivalently 3 mg/kg/day as dosed elsewhere (Malehmir et al., 2019); and 4) HFD + high-dose clopidogrel group: mice fed HFD and drinking bottle water containing clopidogrel of 100 μg/mL, equivalently 10 mg/kg/day for mice or its routine maintenance dose for patient care (Jiang et al., 2022; 2023; Wang et al., 2024). The body weight was measured and recorded for each mouse every Thursday and the dose of clopidogrel was adjusted over time by its body weight.

### 2.2 Measurement of glucose tolerance test (GTT) and insulin tolerance test (ITT)

GTT and ITT were performed at the end of the study, respectively. For GTT assay reflective of insulin secretion, mice were injected i.p. with 1 g/kg glucose after overnight fasting at the 12th week of ND or HFD feeding. For ITT assay indicative of insulin sensitivity, mice were injected i.p. with 0.75 U/kg insulin after fasting for 6 h at the 13th week of ND or HFD feeding. Blood samples were withdrawn from the tail vein at 0, 20, 40, 60, 90, and 120 min after i.p. injection of glucose or insulin, respectively. Blood glucose concentrations were measured using a commercial glucometer (cat #: GA-3; Sinocare Inc., Changsha, Hunan, China) according to the manufacturer’s recommendations, with an area under the blood glucose concentration-time curve (AUC) calculated.

### 2.3 Histological staining and evaluation

After being fasted for 6 h, mice were anesthetized with sodium pentobarbital (70 mg/kg, i.p.) (Laferriere and Pang, 2020), and then euthanized by cervical dislocation at the 14th week of ND or HFD feeding. Liver samples were immediately collected, rinsed in normal saline solution, dried on filter papers, and cut into several parts as required. For histopathological analysis, the liver specimens were fixed in 4% paraformaldehyde, dehydrated, embedded in paraffin, and subsequently sectioned and stained with hematoxylin and eosin (HE). To visualize lipid droplets and size, the frozen liver tissues, embedded by optimal cutting temperature (OCT) compound, were sectioned and then stained with oil red O dye. The histological images of the liver tissues were observed and captured with a light microscope (BX51, Olympus, Tokyo, Japan).

### 2.4 Assay of serum biochemical indices

Blood samples collected from each mouse were kept at ambient temperature for 2 h, and then separated by centrifugation to obtain serum and finally frozen at −80°C until analysis. The serum concentrations of TG, total cholesterol (TC), alanine aminotransferase (ALT), aspartate aminotransferase (AST), high-density lipoprotein cholesterol (HDL-c), and low-density lipoprotein cholesterol (LDL-c) were measured by corresponding commercial assay kits (cat #: A110-1-1 for TG; cat #: A111-1-1 for TC; cat #: C009-2-1 for ALT; cat #: C010-2-1 for AST; cat #: A112-1-1 for HDL-c; and cat #: A113-1-1 for LDL-c) from Jiancheng Bioengineering Institute, Nanjing, Jiangsu, China, according to the manufacturer’s protocols. The intrahepatic levels of TG and TC were measured after the liver tissues had been homogenized with ethanol (see above).

### 2.5 Quantitative reverse-transcription polymerase chain reaction (qRT-PCR) assay

Liver specimens were harvested shortly for extraction of total RNA and subsequent synthesis of complementary DNA (cDNA) according to the protocols supplied by the manufacturers. PCR amplification and melting curve analysis were conducted using ABI 7500 real-time PCR apparatus (Applied Biosystems, Carlsbad, CA, United States) as described elsewhere (Jiang et al., 2022; 2023). The forward and reverse primer sequences for each target gene and the internal reference gene *Actb* (the gene encoding β-actin) were summarized in Supplementary Table S1. The mRNA expression level of a target gene was estimated by a cycle threshold (Ct) value, and its relative expression was presented as fold-change, which was calculated with the following equation: fold-change = 2^−ΔΔCt^, where ΔCt = Ct (target) − Ct (*Actb*).

### 2.6 Western blot assay

After protein extraction of hepatic tissues, the protein expression level of a target protein was measured by Western blot assay as described elsewhere (Jiang et al., 2022; 2023). Briefly, total proteins in liver tissues were extracted with RIPA lysis buffer (cat #: P0013B; Beyotime Biotech, Shanghai, China) supplemented with the inhibitors for both phosphatase and protease (cat #: A32959; Thermo Fisher Scientific, Rockford, IL, United States), and protein concentrations were quantified by BCA protein content kit (cat #: P0011; Beyotime). The following primary antibody was used for the measurement of the target protein in the liver of mice by Western blot assay, and against anti-AMPKα (diluted at 1:1000; cat #: 5831; RRID: AB_10622186; Cell Signaling Technology, Danvers, MA, United States), anti-phospho-AMPKα (Thr172) (1:1000; cat #: 2535; RRID: AB_331250; Cell Signaling Technology), anti-ACC (1:1000; cat #: 3676; RRID: AB_2219397; Cell Signaling Technology), and anti-phospho-ACC (Ser79) (1:1000; cat #: 11818; RRID: AB_2687505; Cell Signaling Technology), and anti-β-actin (1:10000; cat #: 66009-1-Ig; RRID: AB_2687938; Proteintech Group Inc., Chicago, IL, United States). The corresponding secondary antibody used was horseradish peroxidase (HRP)-conjugated anti-mouse (1:10000; cat #: SA00001-1; RRID: AB_2722565; Proteintech) or anti-rabbit (1:10000; cat #: SA00001-2; RRID: AB_2722564; Proteintech). Finally, the immunoreactivity was detected with enhanced chemiluminescence (ECL) system reagent (cat #: P10300; NCM Biotech, Suzhou, Jiangsu, China). The density of each immunoblot band was scanned by the software ImageJ (RRID: SCR_003070; NIH, Bethesda, MD, United States), and a relative expression level of a target protein was normalized to β-actin (as a loading control) in the corresponding sample.

### 2.7 RNA-sequencing of mouse liver tissues

Total RNA was extracted from liver tissue of mice fed ND or HFD alone or in combination with clopidogrel at a high dose for 14 weeks, using TRIzol reagent kit (Invitrogen, Carlsbad, CA, United States) following the manufacturer’s instructions. The quality and integrity of the extracted total RNA were assessed on an Agilent 2100 bioanalyzer (Agilent Technologies, Palo Alto, CA, United States) and detected by RNase-free agarose gel electrophoresis. After enrichment of mRNA by targeted hybridization with oligo (dT) magnetic beads, the enriched mRNA was fragmented into short fragments using fragmentation buffer and reverse-transcribed into cDNA by using NEBNext ultra II RNA library prep kit for Illumina (cat #: E7770, New England Biolabs, Ipswich, MA, United States). Thereafter, the purified double-stranded cDNA products were subjected to PCR amplification, after repairing the end, A-tailing, and ligation to Illumina sequencing adapters. Sequencing of the final cDNA library was performed using Illumina Novaseq 6000 by Gene Denovo Biotechnology Co., Ltd., Guangzhou, China.

To produce clean and high-quality reads, raw reads were further filtered by FASTP (version 0.18.0; R software package; https://www.r-project.org/). All clean reads were aligned with the reference genomes from Ensembl_release111 by HISAT2 (version 2.1.0; R software package). The aligned reads were then assembled using StringTie (version 1.3.1; R software package). The gene expression of each sample was calculated and then converted to a TPM (transcripts per kilobase of exon model per million mapped reads) value using RSEM software (R software package). Differential gene expression analysis between two groups was performed by DESeq2 package (R software package).

A gene with a *p*-value of <0.05 and fold-change of >1.5 is judged as a differentially expressed gene. Only the pathway with its corresponding *p*-value of <0.05 is considered significantly enriched. A gene set with a nominal *p*-value of <0.05 and an FDR (false discovery rate) value of <0.25 is considered statistically significant.

### 2.8 Preparation and treatment of primary mouse hepatocytes

Primary mouse hepatocytes were isolated and prepared using the liver of 8-week-old male C57BL/6J mice using a collagenase perfusion and gradient centrifugation process, as described elsewhere (Charni-Natan and Goldstein, 2020). Briefly, mice were anesthetized and perfused with HBSS solution (cat #: G4203; Servicebio, Wuhan, Hubei, China), followed by the digestion with collagenase buffer (collagenase type IV; cat #: C5138; Sigma) via the portal vein of mice. After that, the liver was dissected and filtered through a 70 μm cell strainer (cat #: abs7232; Absin, Shanghai, China). Subsequently, hepatocytes were isolated by centrifugation at 50 *g* for 5 min twice and viable cells were cultured in the medium of Williams’ Medium E (cat #: 12551-032, Gibco, Grand Island, NY, United States) supplemented with 0.01% L-thyroxine (cat #: T106193; Aladdin, Shanghai, China), 0.01% dexamethasone (cat #: D137736; Aladdin), and 1% penicillin-streptomycin-gentamicin (cat #: C0223; Beyotime) in a 5% CO_2_/water-saturated incubator at 37°C.

To establish a cell model of steatotic liver, primary mouse hepatocytes were challenged with 0.1 mM palmitic acid (PA; cat #: HY-N0830; MCE, Shanghai, China) and 0.2 mM oleic acid (OA; cat #: 0815204; Macklin, Shanghai, China) dissolved in 0.5% fatty acid-free bovine serum albumin (BSA; cat #: B2064; Sigma-Aldrich, St. Louis, MO, United States) for 24 h, using fatty acid-free BSA (0.5%) alone as a vehicle control. Hepatocytes were treated with clopidogrel (cat #: HY-17459; MCE) at 50 μM (dissolved in the vehicle control; see below) or vehicle control containing 0.125% DMSO (cat #: PWL064; Meilunbio, Dalian, China) for 24 h. In addition, to clarify the involvement of the AMPK pathway in the preventive effects of clopidogrel on the hepatic steatosis, hepatocytes were pretreated with the AMPK-specific inhibitor compound C (cat #: HY-13418; MCE) at 5 μM 1 h prior to use of clopidogrel. To observe the extent of intracellular lipid accumulation, cells were fixed with paraformaldehyde, rinsed with isopropanol solution, stained with oil red O dye (cat #: O0625; Sigma-Aldrich) solution, and visualized by a microscope (ECLIPSE Ti, Nikon, Tokyo, Japan).

### 2.9 Statistical analysis

Data reporting and statistical analysis comply with the guidelines on experimental design and data analysis in pharmacology (Lilley et al., 2020; Percie du Sert et al., 2020). Data are presented as mean ± standard deviation (SD), unless otherwise specified. All values were subjected to be normally distributed after being tested by the Shapiro-Wilk test. For the mouse studies, *n* represents the number of individual mice in each group. For the cell studies, *n* represents the number of biological replicates for each group, and therefore, the results shown are derived from at least 3 independent experiments to assure their reproducibility. Statistically significant differences among groups were tested by using unpaired Student’s t*-*test for two-group comparisons, or one-way ANOVA for multiple-group comparisons, followed by Tukey’s *post hoc* test for data showing homogeneity of variance or Tamhane’s T2 (M) *post hoc* test for heteroscedastic data. A difference is considered statistically significant if *p* < 0.05. Statistical analysis was performed using software SPSS 26.0 (RRID: SCR_002865; SPSS Inc., Chicago, IL, United States).

## 3 Results

### 3.1 Clopidogrel ameliorates HFD-induced hepatic steatosis in mice

**FIGURE 1:**
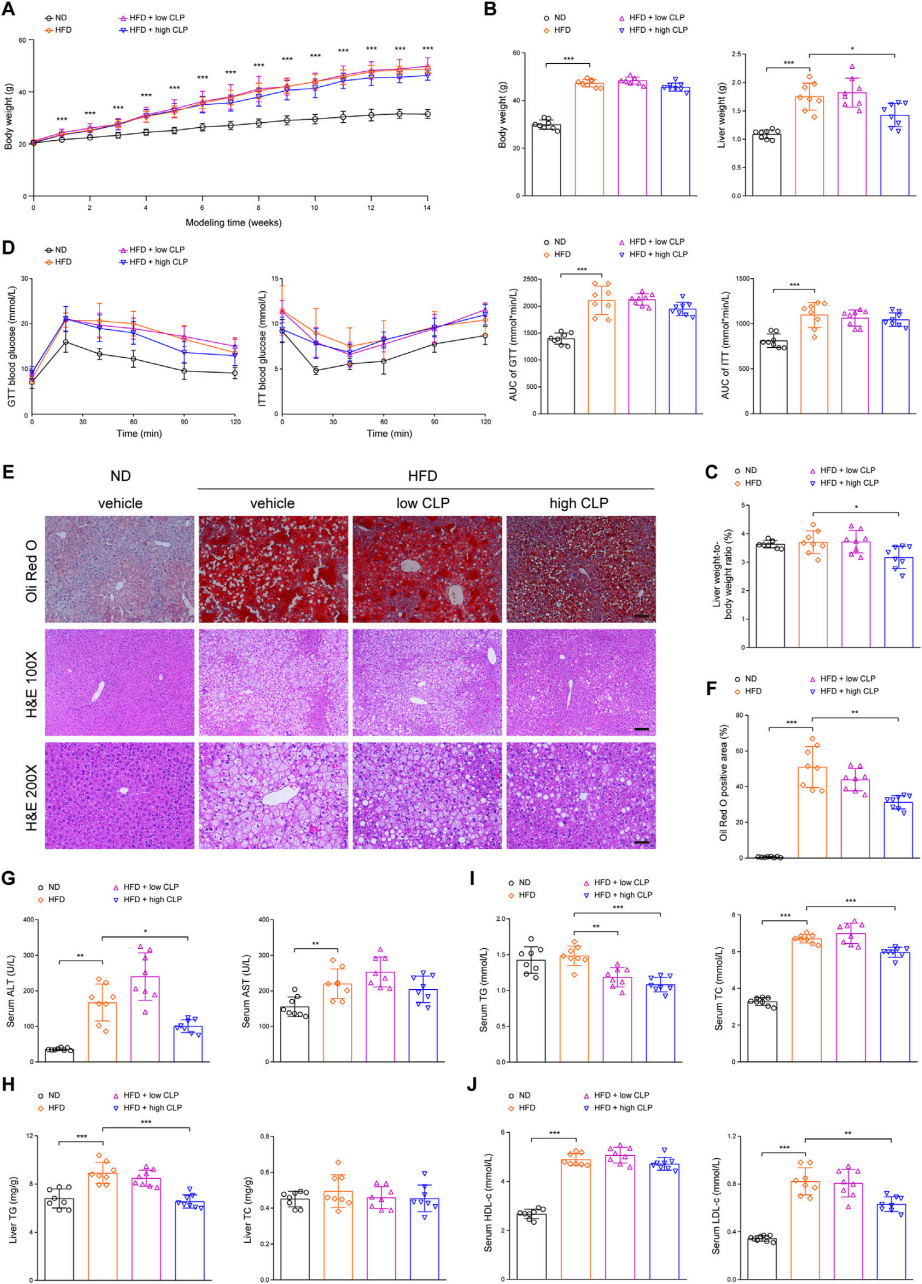
Clopidogrel ameliorates HFD-induced hepatic steatosis in mice. C57BL/6J male mice were fed an ND or an HFD and concomitantly treated with CLP at a low or high dose for 14 weeks (*n* = 8 each). **(A)** Changes in body weight over time of feeding. **(B)** Body weight and liver weight were measured at the end of the study. **(C)** A ratio of liver weight to body weight was measured at the end of the study. **(D)** Blood glucose levels of mice were measured for GTT and ITT, and the corresponding AUC values were calculated, respectively. **(E)** Representative images showing oil red O (upper) and HE (lower) staining of tissue sections of mouse liver (oil red O, scale bar 100 μm; HE 100 ×, scale bar 100 μm; and HE 200 ×, scale bar 50 μm). **(F)** Oil red O staining-positive areas in the liver sections were analyzed and quantified by ImageJ software. **(G)** Serum ALT and AST activity levels of mice. **(H)** Hepatic TG and TC content of mice. **(I)** Serum TG and TC levels of mice. **(J)** Serum HDL-c and LDL-c levels of mice. Data are presented as mean ± SD. *p < 0.05, **p < 0.01, and ***p < 0.001, as indicated. AUC, area under the blood glucose concentration-time curve; ALT, alanine aminotransferase; AST, aspartate aminotransferase; CLP, clopidogrel; GTT, glucose tolerance test; HE, hematoxylin and eosin (staining); HFD, high-fat diet; HDL-c, high-density lipoprotein-cholesterol; ITT, insulin tolerance test; LDL-c, low-density lipoprotein-cholesterol; ND, normal (chow) diet; SD, standard deviation; TC, total cholesterol; TG, triglyceride.

To evaluate the protective effects of clopidogrel on hepatic steatosis, a well-characterized mouse model of HFD-induced hepatic steatosis is a prerequisite to do so. As shown in Figures 1A–D, compared with ND mice, HFD mice exhibited significant elevations in their body weight over time (regardless of the presence or absence of clopidogrel), liver weight, and AUC values of GTT and ITT at the end of 14-week modeling. These data demonstrated that HFD-induced mouse model of hepatic steatosis is characterized by the presence of obesity and insulin resistance, consistent with clinical manifestations of patients with hepatic steatosis (Sergi, 2024). Further evidence that suggested the presence of hepatic steatosis included notable intracellular lipid accumulation and vast fat vacuoles and lipid droplets in the hepatocytes as measured with oil red O staining and presence of more TG-deposited hepatocytes illustrated by HE staining (Figures 1E, F), in parallel with significant elevations in liver TG content as well as serum levels of ALT, AST, TC, HDL-c, and LDL-c (Figures 1G–J). The above data demonstrated the presence of isolated hepatic steatosis (or simple steatosis) in mice after feeding HFD for 14 weeks compared with ND.

To determine the protective effects of clopidogrel on HFD-induced hepatic steatosis in mice, mice received clopidogrel and HFD concomitantly for 14 weeks of the model of hepatic steatosis. When compared with vehicle control, only high-dose clopidogrel (approximately 10 mg/kg/day; equivalent to the clinical maintenance dose of 75 mg/day) significantly reduced liver weight (Figure 1B), the ratio of liver to body weight (Figure 1C), the presence and size of hepatocellular lipid droplets (Figures 1E, F), liver TG content (Figure 1H), serum levels of ALT activity (Figure 1G), a more sensitive biomarker of liver injury than AST (Figure 1G), and serum levels of TG, TC, and LDL-c (Figures 1I, J) at the endpoint time. However, high-dose clopidogrel exerted little or no on the body weight, GTT, ITT, liver TC content, and serum AST levels (Figures 1A, B, D, G, H). In contrast, low-dose clopidogrel (approximately 3 mg/kg/day) failed except for significant serum TG reduction (Figure 1I). These results consistently demonstrated that long-term clopidogrel can effectively prevent mice on HFD feeding from hepatic steatosis when administered at a high dose concomitantly.

### 3.2 Clopidogrel effectively suppresses the MASLD-associated pathways in mice

**FIGURE 2:**
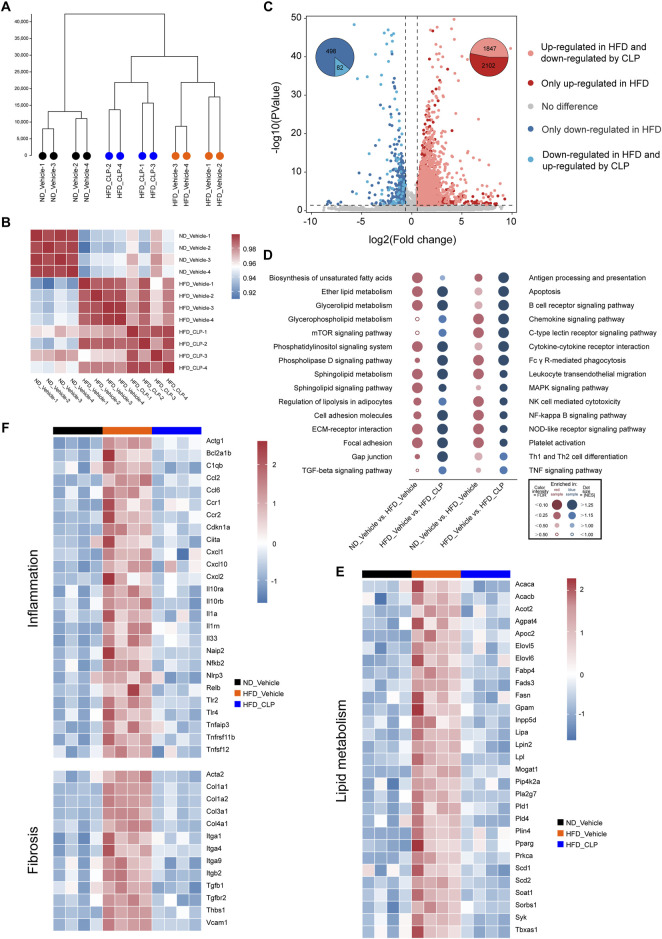
RNA-seq analysis was used to systematically demonstrate the effects of clopidogrel on the expression profiling of genes involved in MASLD. RNA-seq data were acquired from liver tissues of mice, with ND-vehicle, HFD-vehicle, or HFD-CLP categorized (*n* = 4 each). **(A)** Clustering tree illustrating global sample distribution profiles by hierarchical clustering analysis. **(B)** A correlation heatmap showing a correlation between samples by Pearson’s correlation coefficients. **(C)** Volcano plot exhibiting the fold change and *p* values of all genes, using different colors to highlight DEGs of different expression patterns, and using pie charts showing the number of DEGs regulated by HFD feeding and CLP intervention, respectively. **(D)** Dot plot representing pairwise comparisons of GSEA enrichment in KEGG pathways involved in lipid metabolism, inflammation, and fibrosis, all of which were upregulated by HFD feeding but downregulated by CLP intervention. Heat maps displaying different expression patterns of representative DEGs involved in lipid metabolism **(E)**, inflammation and fibrosis **(F)** that were upregulated by HFD feeding but reversed by CLP. DEG, differentially expressed gene; FDR, false discovery rate; GSEA, gene set enrichment analysis; KEGG, Kyoto Encyclopedia of Genes and Genomes; NES, normalized enrichment score. Other abbreviations, see those shown in Figure 1.

To systematically decipher the mechanisms of how clopidogrel protects mice from HFD-induced hepatic steatosis, transcriptomics (also known as RNA-seq) analysis was conducted for the liver samples acquired from mice on ND-vehicle, HFD-vehicle, or HFD-clopidogrel (CLP) (as shown in Figure 2). Unsupervised hierarchical clustering analysis showed that there was strong consistency in gene expression profiles within each of the three groups, and that ND-vehicle, HFD-vehicle, and HFD-CLP samples were clearly separated each other, especially ND-vehicle vs HFD-vehicle groups, confirming that the construction of the HFD-induced hepatic steatosis model was successful as anticipated, which is crucial for the subsequent investigations of changes in the molecular mechanisms and pathways (Figure 2A). Next, a correlation heatmap analysis revealed that, compared with the gene expression patterns of HFD-vehicle, those of HFD-CLP exhibited a more pronounced correlation with those of ND-vehicle, as indicated by the deeper red and lighter blue coloration in the heatmap, suggesting the potential of clopidogrel in modulating HFD-induced dysregulations in the signaling pathways involved (Figure 2B). Of the differentially expressed genes observed in the liver between ND-vehicle and HFD-vehicle groups, 3949 genes were upregulated, and 580 were downregulated by HFD feeding, as shown in the volcano plot (Figure 2C). Further, of the HFD-modulated genes, 1847 upregulated and 82 downregulated genes were all reversed by clopidogrel (Figure 2C). Gene set enrichment analysis (GSEA) indicated that genes affected by HFD feeding were enriched for the pathways that are associated with the progression of MASLD, including but not limited to lipid metabolic processes (e.g., ether lipid metabolism, glycerolipid metabolism, and sphingolipid metabolism), inflammatory responses (e.g., chemokine signaling pathway, C-type lectin receptor signaling pathway, and NK cell-mediated cytotoxicity), and fibrotic pathways (e.g., cell adhesion molecules, ECM-receptor interactions, and focal adhesion), all of which were significantly suppressed by clopidogrel (Figure 2D). In addition, RNA-seq results showed that the expression levels of genes related to lipid metabolism, inflammation, and fibrosis were significantly upregulated by HFD feeding but downregulated by clopidogrel, as depicted in the heatmap (see Figures 2E, F). All the results demonstrated that HFD feeding significantly upregulates the genes involved in lipid metabolism, inflammation, and fibrosis processes of mouse hepatocytes, but that clopidogrel effectively attenuates such abnormal changes in HFD-fed mice.

### 3.3 Clopidogrel attenuates HFD-induced aberrant lipid metabolism by enhanced phosphorylation of the AMPK signaling pathway in mice

**FIGURE 3:**
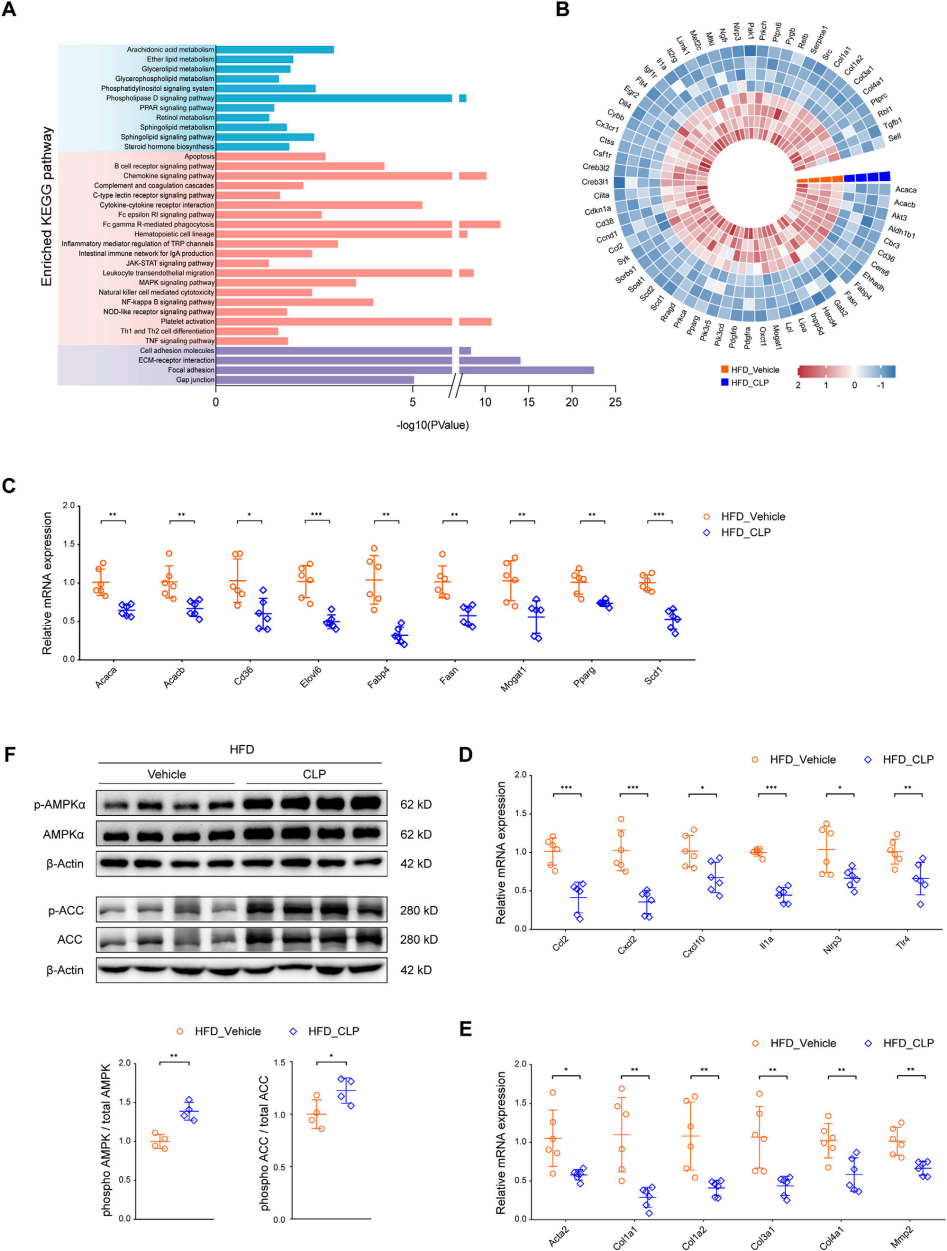
Clopidogrel activates the AMPK pathway in mice. **(A)** KEGG pathway enrichment analysis showing the pathways involved in lipid metabolism, inflammation, and fibrosis, all of which were regulated by clopidogrel in HFD-fed mice (*n* = 4 each). **(B)** A heatmap illustrating the expression profiles of genes responsible for lipid metabolism, inflammation, and fibrosis present in the liver of mice on either HFD-vehicle or HFD-CLP, which was associated with differentially phosphorylated AMPK signaling molecules based on STRING and PubMed database (*n* = 4 each). qRT-PCR analysis demonstrating relative mRNA expression levels of **(C)** lipid metabolism-related genes (*Acaca, Acacb*, *Cd36*, *Elovl6*, *Fabp4*, *Fasn*, *Mogat1*, *Pparg*, and *Scd1*), **(D)** proinflammatory genes (*Ccl2*, *Cxcl2*, *Cxcl10*, *Il1a, Nlrp3,* and *Tlr4*), and **(E)** profibrotic genes (*Acta2*, *Col1a1*, *Col1a2*, *Col3a1*, *Col4a1*, and *Mmp2*) in the liver of mice on either HFD-vehicle or HFD-CLP (*n* = 6 each). **(F)** Western blot assay of total and phosphorylated AMPKα and ACC in the liver of mice on HFD-vehicle or HFD-CLP (*n* = 4 each). β-Actin was used as a loading control for all cell lysates. Each target protein was quantified by ImageJ software. Other abbreviations, see those shown in Figures 1, 2.

A comprehensive analysis of KEGG (Kyoto Encyclopedia of Genes and Genomes) pathway enrichment data (acquired via RNA-seq) uncovered that clopidogrel can modulate the signaling pathways essential for lipid metabolism, inflammatory responses, and fibrotic processes in mice on HFD (Figure 3A). Next, thorough analyses of the expression profiles of the genes responsible for these processes revealed that enhanced phosphorylation of AMPK and its downstream signaling protein(s) were correlated with the downregulation of genes mediated by CLP according to the STRING and PubMed databases (Figure 3B). Thereafter, qRT-PCR assays were used to further validate the main results derived from RNA-seq data. As expected, clopidogrel downregulated the expression of genes responsible for fatty acid synthesis (*Acaca*/*Acacb*, *Elovl6*, *Fasn*, *Mogat1*, and *Scd1*), fatty acid uptake (*Cd36*, *Fabp4*, and *Pparg*), proinflammatory cytokines (*Ccl2*, *Cxcl2*, *Cxcl10*, *Il1a*, *Nlrp3*, and *Tlr4*), as well as fibrogenesis (*Acta2*, *Col1a1*, *Col1a2*, *Col3a1*, *Col4a1*, and *Mmp2*) in the liver of HFD-fed mice (Figures 3C–E).

Considering the well-recognized involvement of AMPK in the pathogenesis and progression of MASLD, we hypothesized that the protective effects of clopidogrel on HFD-triggered abnormal lipid metabolism in mouse liver might be mediated primarily through activation of AMPK. To test this hypothesis, Western blot assay was used to evaluate changes in the phosphorylation levels of AMPK and its downstream acetyl coenzyme A carboxylase (ACC, encoded by *Acaca* and *Acacb* in mice) (Figure 3F). Consistent with our expectations, clopidogrel conferred a marked elevation in the phosphorylation level of AMPK in the liver of HFD-fed mice. Moreover, the phosphorylation level of ACC was enhanced by clopidogrel.

### 3.4 Clopidogrel suppresses fatty acid synthesis of hepatocytes *in vitro* through enhanced phosphorylation of the AMPK pathway

**FIGURE 4:**
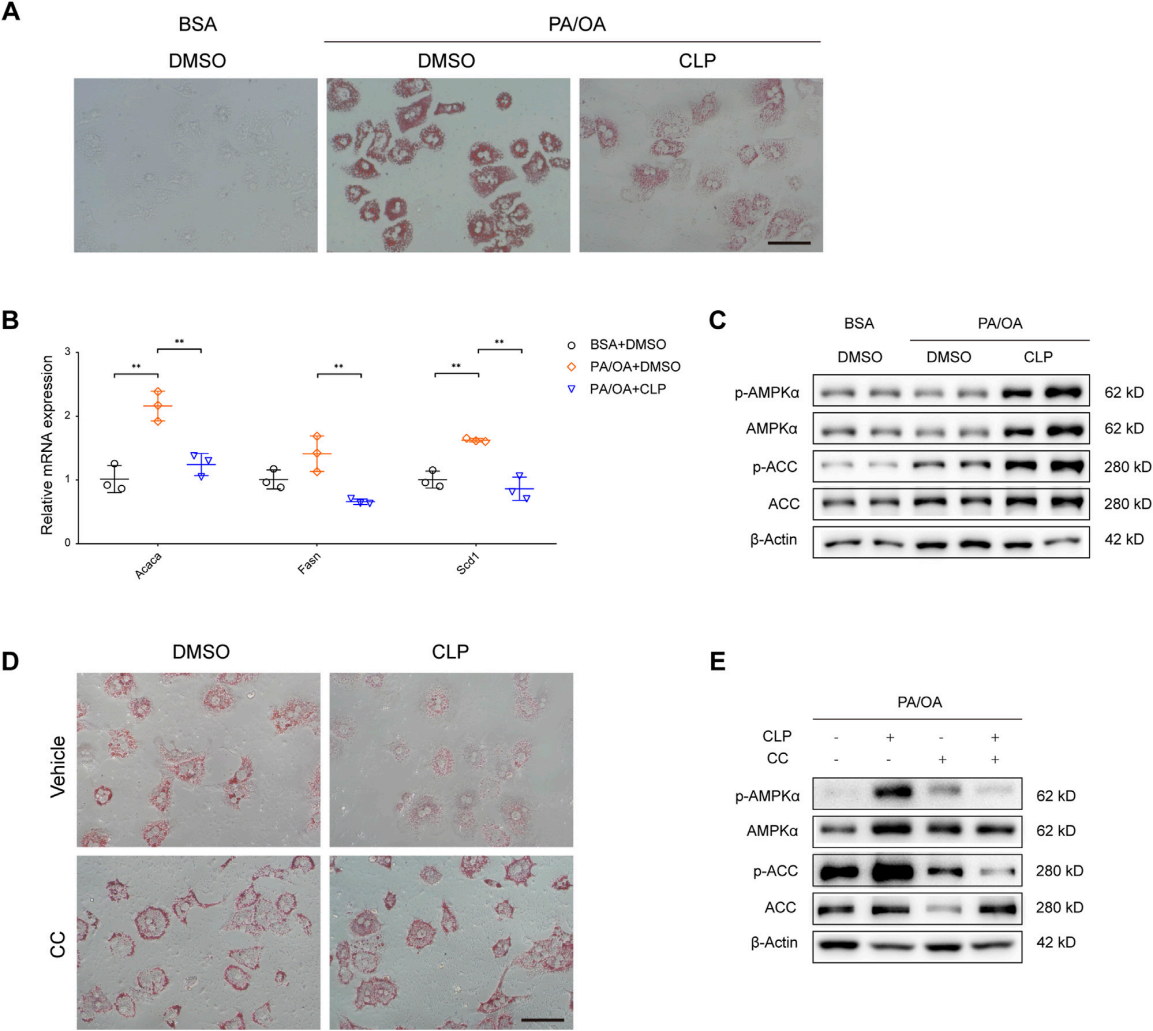
Clopidogrel reduces lipid accumulation by enhanced phosphorylation of the AMPK pathway in mouse primary hepatocytes. **(A–C)** Mouse primary hepatocytes were treated with BSA or a combination of palmitic acid (PA) and oleic acid (OA) (PA/OA, 0.3 mM), and then treated with DMSO or CLP (50 μM) for 24 h. **(A)** Representative images showing oil red O staining of mouse primary hepatocytes. Scale bar, 100 μm. **(B)** qRT-PCR analysis showing relative mRNA expression levels of genes responsible for lipid metabolism (*Acaca*, *Fasn*, and *Scd1*) in mouse primary hepatocytes as indicated, whose mRNA expression was normalized to that of *Actb*. **(C)** Western blot assay of total and phosphorylated AMPKα and ACC in primary mouse hepatocytes. β-Actin was used as a loading control for all cell lysates. **(D, E)** Primary mouse hepatocytes were challenged by PA/OA (0.3 mM) and treated with CLP (50 μM) and/or Compound C (CC, 5 μM) for 24 h. **(D)** Representative images showing oil red O staining of primary mouse hepatocytes. Scale bar, 100 μm. **(E)** Western blot assay of total and phosphorylated AMPKα and ACC in primary mouse hepatocytes. β-Actin was used as a loading control for all cell lysates. Results shown are representative of at least three independent experiments. ACC, acetyl-CoA carboxylase; *Actb*; gene encoding β-actin; AMPK, AMP-activated protein kinase; BSA, bovine serum albumin; CC, compound C; DMSO, dimethyl sulfoxide; PA/OA, palmitic acid and oleic acid. Other abbreviations, see those shown in Figures 1–3.

Given that hepatocytes are the principal parenchymal cells of the liver and play a crucial role in mediating lipotoxicity triggered by fatty acids, we proceeded to examine the direct effects of clopidogrel on hepatocytes exposed to metabolic stress. To explore the *in vitro* therapeutic potential of clopidogrel, primary mouse hepatocytes were challenged by PA and OA and treated with clopidogrel for 24 h. Oil red O staining results revealed that when exposed to PA/OA, primary mouse hepatocytes exhibited a pronounced increase in intracellular lipid accumulation as evidenced with presence of more oil red-stained lipid droplets than BSA-treated controls (Figure 4A). Clopidogrel intervention led to reduced lipid accumulation in PA/OA-stimulated hepatocytes as shown by a comparatively smaller cluster of lipid droplets within hepatocytes when compared with DMSO control (Figure 4A). Similarly, qRT-PCR analysis showed that PA/OA-induced upregulation of the lipogenic genes (such as *Acaca*, *Fasn*, and *Scd1*) was attenuated by clopidogrel effectively (Figure 4B). Consistent with the above, after exposure to PA/OA alone or in combination with clopidogrel, primary mouse hepatocytes exhibited greater phosphorylation levels of AMPKα and ACC by clopidogrel than by vehicle control as measured by Western blot assay (Figure 4C).

To further verify whether AMPK activation could mediate inhibitory effects of clopidogrel on intracellular lipid accumulation, primary mouse hepatocytes were exposed to compound C (an AMPK-specific inhibitor (Quan et al., 2013; Yang et al., 2016) under PA/OA challenge and in the presence or absence of clopidogrel (Figures 4D, E). As shown in Figure 4D, the accumulation of lipids in PA/OA-treated primary mouse hepatocytes was significantly diminished by clopidogrel; however, use of compound C abolished the beneficial effect of clopidogrel in mitigating PA/OA-induced lipid deposition, concomitant with reversal of the enhanced phosphorylation levels of AMPKα and ACC induced by clopidogrel (Figure 4E). These results clearly demonstrated that AMPKα (if activated) plays an indispensable role in the observed protective effects of clopidogrel on PA/OA-treated primary mouse hepatocytes, and that clopidogrel ameliorates lipid accumulation in this cell model through enhanced phosphorylation of the AMPK/ACC signaling pathway.

## 4 Discussion

In this work, we used HFD-induced obese mice to investigate the effects of clopidogrel on the initiation and progression of hepatic steatosis and its potential mechanisms and observed that clopidogrel ameliorates HFD-induced hepatic steatosis in obese mice by attenuation of aberrant fatty acid *de novo* biosynthesis via the multiple MASLD-associated pathways, or primarily through enhanced phosphorylation of the AMPK/ACC pathway. This study provided consistent evidence *in vivo* and *in vitro* for the beneficial (preventive and/or therapeutic) effects of clopidogrel on hepatic steatosis in HFD-induced obese mice, with a major focus on its systems pharmacology exploration.

There have been multiple animal models that are used to investigate the pathogenesis of MASLD and its therapeutic strategies. Of them, HFD-induced obese mouse model is closer to human MASLD status, characterized by obesity (body weight gain), insulin resistance, intracellular TG accumulation in the liver, hepatocellular fat vacuoles, and elevations in serum levels of ALT, AST, TC, and LDL-c as shown in this study. As expected, compared with vehicle control, clopidogrel exerted significant preventive effects on steatotic liver in HFD-fed mice, as demonstrated by marked decreases in hepatocyte size and intrahepatocellular lipid droplet size and TG-deposited hepatocytes, and consequently, pronounced decreases in liver weight and a ratio of liver weight to body weight. Because mice received clopidogrel and HFD concomitantly for 14 weeks of the entire model construction, clopidogrel may also possess the potential therapeutic effects on steatotic liver at the late stage of that model construction in addition to the primary prevention against that diseased status at its early stage.

From the transcriptomic sequencing data of the liver tissues of mice fed ND or HFD alone or in combination with clopidogrel, three main categories of differentially expressed genes were enriched as lipid metabolism, inflammation, and fibrosis according to clustering or patterns of their expression. Of the clustering of the lipid metabolism-related genes, both *Acaca* and *Acacb* code for AAC (downstream of SERBP-1c if phosphorylated), *Fasn* codes for Fasn (fatty acid synthase), *Scd1* codes for Scd1 (stearoyl-CoA desaturase, synthesizing oleate), *Elovl6* codes for Elovl6 (elongase of very long chain fatty acids 6, synthesizing palmitate), and *Mogat1* codes for Mogat1 (monoacylglycerol *O*-acyltransferase 1, synthesizing diacylglycerol), all of which constitute a common signaling pathway – AAC/Fasn/Scd1/Elovl6/Mogat1 – as descried elsewhere (Lin et al., 2024; Liss et al., 2021; Moon et al., 2014; Tao et al., 2024; Zhu et al., 2019), which is downstream of the AMPK pathway that is responsible for the *de novo* biosynthesis of fatty acids (Hong et al., 2020; Lee et al., 2023; Ma et al., 2012; Xi et al., 2015; Zhang et al., 2024; Zhu et al., 2018; 2019). Further, compared with vehicle control, clopidogrel increased the phosphorylation levels of AMPKα and ACC in the liver of HFD-fed mice and in PA/OA challenged primary mouse hepatocytes, but clopidogrel-induced elevation of these phosphorylation was reversed by compound C (an AMPK inhibitor) (Quan et al., 2013; Yang et al., 2016) in primary mouse hepatocytes. Clearly, compared with regular ND, HFD significantly upregulated the lipogenic genes responsible for the biosynthesis of fatty acids in the liver, but clopidogrel reversed such upregulation, suggesting that clopidogrel may protect mice against HFD-induced hepatic steatosis through enhanced phosphorylation of the AMPK signaling pathway, consistent with a previous finding that clopidogrel activates AMPK (Yang et al., 2016), as do other candidate compounds for MASLD, including but not limited to metformin (Zhu et al., 2018), berberine (Zhu et al., 2019), resveratrol (Trepiana et al., 2018), baicalin (Ma et al., 2012; Xi et al., 2015), and betulinic acid (Quan et al., 2013). Emerging evidence has been demonstrated that AMPK serves as one of the important central controllers of the cellular energy metabolism to achieve the balance between catabolism and anabolism in two ways: quickly modulating the function and position of crucial metabolic enzymes by direct phosphorylation, and gradually regulating the expression of various gene sets through transcriptional response that takes longer to manifest (Ahmad et al., 2020; Mihaylova and Shaw, 2011). For example, AMPK suppresses the activation of Srebp1 through phosphorylating it and reduces ACC activity also through phosphorylating ACC1 and ACC2, where Srebp1 induces its downstream effectors (primarily ACC1 and Fasn) that accelerate fatty acid synthesis *de novo*, and reduction of ACC activity will decrease malonyl-CoA and lead to the inhibition of *de novo* lipogenesis and increase fatty acid oxidation in the cellular mitochondria (Ahmad et al., 2020; Mihaylova and Shaw, 2011). In this study, clopidogrel was found to be an AMPK activator, exerting its inhibitory effects on lipogenesis *de novo*.

Hepatocytes not only synthesize fatty acids *de novo*, but also import exogenous fatty acids from diet. Earlier studies demonstrated that fatty liver exhibited elevation of *Pparg* expression and conversely, ablation of liver *Pparg* led to reductions in hepatic steatosis (Gavrilova et al., 2003) and hepatic TG content through downregulation of hepatic lipogenic genes (*Fasn*, *Acaca*/*Acacb*, and *Scd1*) (Matsusue et al., 2003), that use of rosiglitazone (a Pparg activator) exacerbated hepatic steatosis through activating genes responsible for lipid uptake such as *Cd36* (encoding cluster of differentiation 36) and *Fabp4* (encoding fatty acid binding protein 4) (Ahmadian et al., 2013; García-Ruiz et al., 2007; Matsusue et al., 2003), but that absence of hepatic *Pparg* worsened hyperlipidemia and TG clearance (Gavrilova et al., 2003). These results demonstrated that hepatic Pparg modulates TG homeostasis, contributing to hepatic steatosis, but protects other extrahepatic tissues (such as muscle) from TG accumulation and insulin resistance (Ahmadian et al., 2013; García-Ruiz et al., 2007; Gavrilova et al., 2003; Matsusue et al., 2003). Consistent with the above findings, compared with vehicle control, clopidogrel downregulated the expression of liver *Pparg*, and thus suppressed the expression of hepatic lipogenic genes (*Fasn*, *Acaca*/*Acacb*, and *Scd1*) and genes involved in fatty acid trafficking/uptake (*Cd36* and *Fabp4*), all of which are completely consistent with their corresponding changes in the suppression or deficiency of *Pparg* as reported elsewhere (Ahmadian et al., 2013; García-Ruiz et al., 2007; Gavrilova et al., 2003; Matsusue et al., 2003). In addition, this study showed that clopidogrel alleviated hepatic steatosis, probably through restraining CD36-mediated fatty acid uptake, consistent with a previous finding that clopidogrel downregulates the expression of CD36 (Mokgalaboni et al., 2020). These data suggested that clopidogrel may be a new pan (or multifunctional) inhibitor of Pparg, CD36, and Fabp4, in a direct or indirect manner.

On the other hand, low-grade inflammation runs through the entire process of MASLD, with fibrosis occurring at a late stage. As expected, in this study, multiple key genes encoding proinflammatory cytokines and chemokines, including but not limited to *Ccl2*, *Cxcl2*, *Tlr4*, and *Nlrp3*, were expressed differentially across the three groups of mice fed ND or HFD or HFD in combination with clopidogrel. Clopidogrel significantly downregulated the mRNA expression of multiple proinflammatory cytokines and chemokines in HFD-fed mice, which is consistent with earlier findings that clopidogrel exerts systemic anti-inflammatory effects through suppression of platelet activation or exerting its pleiotropic effects as reported elsewhere (Cerda et al., 2017; Graff et al., 2005; Hadi et al., 2013; Li et al., 2007). Although fibrosis may occur at a relatively late stage of the MASLD process, 14-week HFD feeding led to fatty liver (predominantly hepatic steatosis), where several pro-fibrotic genes (including *Acta2*, *Col1a1*, *Col1a2*, *Col3a1*, *Col4a1*, and *Mmp2*) (Fernández-Ginés et al., 2024; Wu et al., 2024) and 3 out of 12 identified critical hub genes of fibrosis (such as *Col1a1*, *Col1a2*, and *Col3a1*) (Mahmoudi et al., 2024) were upregulated by HFD, but were reversed by clopidogrel, which is consistent with the reports of others that clopidogrel may exert anti-fibrotic effects, such as suppression of α-SMA (a well-characterized fibrosis marker (Pivovarova-Ramich et al., 2021) by clopidogrel in atherosclerotic plaques in *ApoE* ^−/−^ mice fed HFD (Chen et al., 2020).

However, there are limitations to this study that need to be addressed further. First, there are multiple animal disease models of MASLD, of which only HFD-fed mouse model was used for this study, because it is considered as one closer to human MASLD. Second, 14-week HFD feeding causes fatty liver (primarily hepatocyte steatosis), and thus, all acquired conclusions in this study cannot be extrapolated to other stages of MASLD process. Third, male mice may be more susceptible to fatty liver (Gallage et al., 2022), and only male mice were used in this study. Therefore, interpretation of its results should be with caution for female mice. Fourth, species differences exist between mice and human beings, and the generalizability of these findings should be validated in human subjects. Last, this study was a primary investigation about whether clopidogrel could prevent mice from HFD-induced hepatic steatosis and how it works. Further validation studies will be required in the future.

In summary, this study provided substantial experimental evidence that clopidogrel can prevent and reverse HFD-induced hepatic steatosis in mice through enhanced phosphorylation of the AMPK signaling pathway and suppression of PPARG-mediated uptake of fatty acids in the liver and primary hepatocytes of mice, suggesting that clopidogrel could be repurposed to fight MASLD for future patient care.

## Data Availability

The raw sequence data reported in this paper have been deposited in the Genome Sequence Archive (Chen et al., 2021) in National Genomics Data Center (CNCB-NGDC Members and Partners, 2022), China National Center for Bioinformation/Beijing Institute of Genomics, Chinese Academy of Sciences (GSA: CRA019070), which are publicly accessible at https://ngdc.cncb.ac.cn/gsa.
