# Supplementary material for: Clopidogrel ameliorates high-fat diet-induced hepatic steatosis in mice through activation of the AMPK signaling pathway and beyond
Source: Front Pharmacol. 2024 Oct 23;15:1496639. doi: 10.3389/fphar.2024.1496639 (PMC11537861; doi:10.3389/fphar.2024.1496639)
Supplement: Supplementary file 1 [file Table1.docx]

**Supplementary Table 1. The primers used for quantitative real-time PCR analysis of genes in mice.**

| **Gene** |  | **Sequence (5’ → 3’)** |
| --- | --- | --- |
| *Acaca* | F | CCCAGAGATGTTTCGGCAGTCAC |
|  | R | GTCAGGATGTCGGAAGGCAAAGG |
| *Acacb* | F | TGAATCTCACGCGCCTACTATG |
|  | R | ATGACCCTGTTGCCTCCAAAC |
| *Acta2* | F | AGCCATCTTTCATTGGGATGG |
|  | R | CCCCTGACAGGACGTTGTTA |
| *Actb* | F | GTACCACCATGTACCCAGGC |
|  | R | AACGCAGCTCAGTAACAGTCC |
| *Ccl2* | F | CCACAACCACCTCAAGCACT |
|  | R | TAAGGCATCACAGTCCGAGTC |
| *Cd36* | F | CTTTGAAAGAACTCTTGTGGGG |
|  | R | GTCTGTGCCATTAATCATGTCG |
| *Cxcl10* | F | ATGACGGGCCAGTGAGAATG |
|  | R | ATGATCTCAACACGTGGGCA |
| *Cxcl2* | F | CCAACCACCAGGCTACAGG |
|  | R | GCGTCACACTCAAGCTCTG |
| *Col1a1* | F | TCCTCCAGGGATCCAACGA |
|  | R | GGCAGGCGGGAGGTCTT |
| *Col1a2* | F | ATCCGGTAACAAGGGTGAGC |
|  | R | GAACCAGGGCTGCCTCTAAG |
| *Col3a1* | F | CTGGTCAGCCTGGAGATAAG |
|  | R | ACCAGGACTACCACGTTCAC |
| *Col4a1* | F | CTTTGAAAGAACTCTTGTGGGG |
|  | R | GTCTGTGCCATTAATCATGTCG |
| *Elovl6* | F | TGCCATGTTCATCACCTTGT |
|  | R | TGCTGCATCCAGTTGAAGAC |
| *Fabp4* | F | TGAAATCACCGCAGACGACAGG |
|  | R | GCTTGTCACCATCTCGTTTTCTC |
| *Fasn* | F | AAGTTGCCCGAGTCAGAGAA |
|  | R | CGTCGAACTTGGAGAGATCC |
| *Il1a* | F | GCGATACACTCTGGTGCTCA |
|  | R | CCCAGGGAAACCAGGATATT |
| *Mmp2* | F | GCTGATACTGACACTGGTACTG |
|  | R | CAATCTTTTCTGGGAGCTC |
| *Mogat1* | F | GCCAGTTTGGTTCCAGTATTTT |
|  | R | CGTCTTGTATAGTTCGTAGCCA |
| *Nlrp3* | F | CAAGGCTGCTATCTGGAGGAAC |
|  | R | TCGCAGCAAAGATCCACACA |
| *Pparg* | F | CCACAGTTGATTTCTCCAGCATTTC |
|  | R | CAGGTTCTACTTTGATCGCACTTTG |
| *Scd1* | F | GCGATACACTCTGGTGCTCA |
|  | R | CCCAGGGAAACCAGGATATT |
| *Tlr4* | F | GTTCTCTCATGGCCTCCACT |
|  | R | AGGGACTTTGCTGAGTTTCTGAT |

F, forward; R, reverse.
